# AntiRef: reference clusters of human antibody sequences

**DOI:** 10.1093/bioadv/vbad109

**Published:** 2023-08-22

**Authors:** Bryan Briney

**Affiliations:** Department of Immunology and Microbiology, The Scripps Research Institute, La Jolla, CA 92037, United States; Center for Viral Systems Biology, The Scripps Research Institute, La Jolla, CA 92037, United States; The Multiomics Vaccine Evaluation Consortium, The Scripps Research Institute, La Jolla, CA 92037, United States; Scripps Center for HIV/AIDS Vaccine Development, The Scripps Research Institute, La Jolla, CA 92037, United States; San Diego Center for AIDS Research, The Scripps Research Institute, La Jolla, CA 92037, United States

## Abstract

**Motivation:**

Genetic biases in the human antibody repertoire result in publicly available antibody sequence datasets that contain many duplicate or highly similar sequences. Available datasets are further skewed by the predominance of studies focused on specific disease states, primarily cancer, autoimmunity, and a small number of infectious diseases that includes HIV, influenza, and SARS-CoV-2. These biases and redundancies are a barrier to rapid similarity searches and reduce the efficiency with which these datasets can be used to train statistical or machine-learning models. Identity-based clustering provides a solution; however, the extremely large size of available antibody sequence datasets makes such clustering operations computationally intensive and potentially out of reach for many scientists and researchers who would benefit from such data.

**Results:**

Antibody Reference Clusters (AntiRef), which is modeled after UniRef, provides clustered datasets of filtered human antibody sequences. Due to the modular nature of recombined antibody genes, the clustering thresholds used by UniRef for general protein sequences are suboptimal for antibody clustering. Starting with an input dataset of ∼451M full-length, productive human antibody sequences, AntiRef provides reference datasets clustered at a range of antibody-optimized identity thresholds. AntiRef90 is one-third the size of the input dataset and less than half the size of the non-redundant AntiRef100.

**Availability and implementation:**

AntiRef datasets are available on Zenodo (zenodo.org/record/7474336). All code used to generate AntiRef is available on GitHub (github.com/briney/antiref). The AntiRef versioning scheme (current version: v2022.12.14) refers to the date on which sequences were retrieved from OAS.

## 1 Introduction

The massive diversity of the human antibody (Ab) repertoire is produced initially by somatic recombination of germline gene segments. Considering both heavy and light chains, it is estimated that the recombination process can generate as many as 1018 unique Abs (Briney *et al.* 2019). For perspective, this surpasses the combined number of unique proteins encoded by all the genomes of all species on earth by many orders of magnitude (Mora *et al.* 2011). High-throughput genetic analysis of Ab repertoires became technically possible with read-length enhancements on the 454 GS-FLX in 2009 (Weinstein *et al.* 2009). Over the ensuing decade, next-generation sequencing technology has markedly improved (Finn and Crowe 2013), enabling the first ultra-deep analyses of the human Ab repertoire using billions of sequencing reads (Briney *et al.* 2019, Soto *et al.* 2019). These increasingly large Ab repertoire datasets have been reused in a variety of novel ways, including mining naturally occurring repertoires for homologs to therapeutic antibodies and uncovering vaccine-targetable precursors of exceptionally broad antiviral antibodies (Jardine *et al.* 2016, Krawczyk *et al.* 2019, Steichen *et al.* 2019, Hurtado *et al.* 2022). One of the most exciting areas of emerging research is the development of sophisticated machine-learning models of antibodies and Ab repertoires. Following in the footsteps of large language models for text (Devlin *et al.* 2018, Brown *et al.* 2020) and general protein sequences (Lin *et al.* 2022), Ab-specific language models have begun learning features unique to antibodies (Leem *et al.* 2021, Ruffolo *et al.* 2021, Olsen *et al.* 2022b) and can be fine-tuned to perform downstream tasks, such as structure and paratope prediction (Leem *et al.* 2021, Ruffolo *et al.* 2022).

These studies are hampered, by the scale of available Ab sequence data and the lack of standardized datasets of substantially reduced size that maintain an accurate representation of overall diversity. The availability of such datasets is vitally important; one particularly relevant recent example is the use of UniRef datasets (Suzek *et al.* 2007, 2015) to train the state-of-the-art protein language model ESM-2 (Lin *et al.* 2022). We lack a “UniRef for antibodies” in part because its creation is sufficiently computationally intensive to be infeasible for many who would nevertheless benefit from such a dataset. Here, we present AntiRef, a UniRef-inspired, standardized dataset of clustered human Ab sequences.

## 2 Methods

The Ab sequences used to construct AntiRef were downloaded from the Observed Antibody Space (OAS) repository (Kovaltsuk *et al.* 2018, Olsen *et al.* 2022a). Sequences were filtered using OAS’s query tool prior using the following criteria:

- *Species*: Human
- *BSource*: PBMC
- *Disease*: None
- *Vaccine*: None.

All sequences that met these criteria were retrieved using the download files generated by OAS. After download, sequences were further filtered to retain only sequences with: (i) a complete VDJ region, (ii) no V-gene frameshifts, (iii) in-frame V and J genes, (iv) no stop codons, and (v) no ambiguous amino acids. After filtering, the dataset contained 260 373 862 heavy chains and 190 684 852 light chains, for a total of 451 058 708 sequences. Each sequence was given a unique identifier using Python’s built-in uuid.uuid4() function.

Filtered sequences were iteratively clustered using the linclust function in MMseqs2 (Steinegger and Söding 2018). Clustering operations were ordered such that the identity threshold decreased with each round. This clustering approach was selected because the runtime of linclust scales linearly with input dataset size and because MMseqs2 provides tools for updating a previously clustered dataset with additional input data without re-clustering the entire dataset from scratch. Clustering was performed using amino acid sequences, and clustering operations were ordered such that the identity threshold decreased with each round. Clustering was performed using the following runtime options: (i) -c 0.8 and –cov-mode 0, which together require that linked sequences must align for at least 80% of the length of the longer sequence; and (ii) –min-seq-id <threshold>, which clusters sequences using the appropriate identity threshold for each AntiRef clustering operation. The sequence identity is computed across the entire aligned region, including gaps, meaning sequences that are otherwise identical but differ in length by *N* amino acids would be scored equivalently to two sequences of the same length with *N* mismatches. Following each iteration, representative sequences for each cluster were used as input for the subsequent round. This mirrors the strategy used by UniRef and ensures continuity of cluster names across all AntiRef datasets (Suzek *et al.* 2007).

## 3 Results

### 3.1 Database size reduction

**Figure 1.**
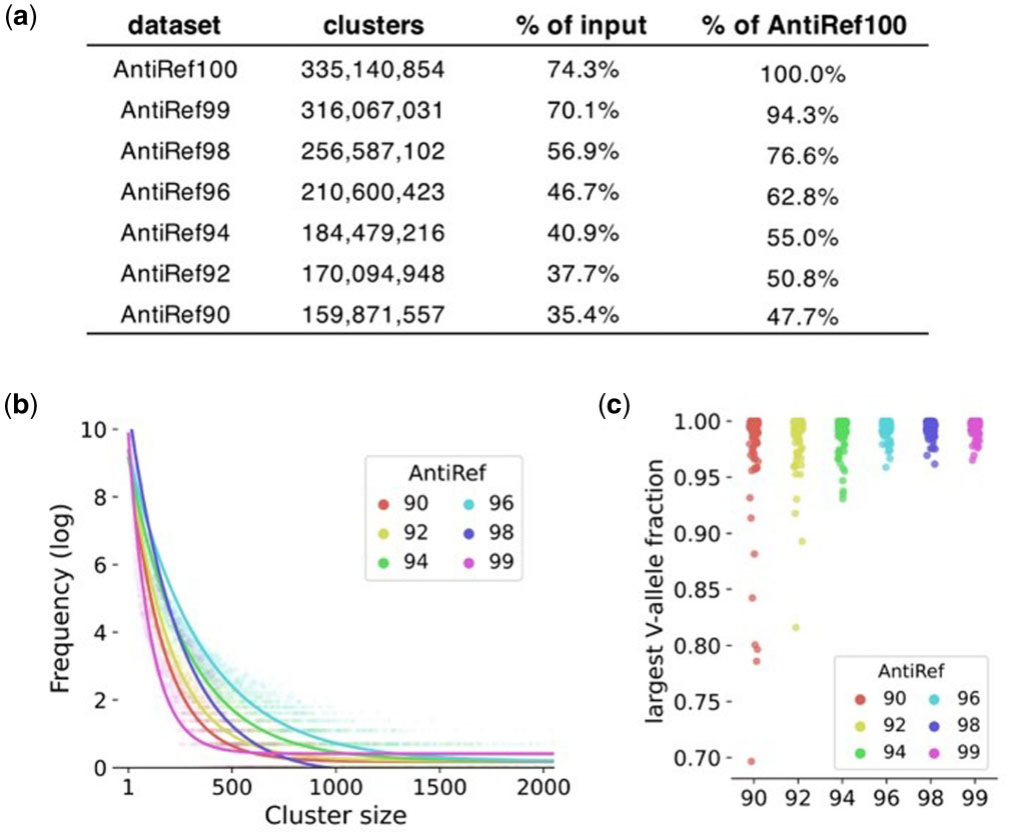
Properties of AntiRef clusters. (a) Size of each AntiRef dataset, including the reduction in size compared to the non-redundant input dataset (AntiRef100). (b) Cluster sizes were computed for each AntiRef dataset, and the frequency of each cluster size is plotted. The best fit line of the cluster size frequency distribution was separately computed for each AntiRef dataset in Python using scipy.optimize.best_fit(). The cluster size frequencies follow a power law distribution. (c) For the largest 100 clusters in each AntiRef dataset, the relative fraction of the most common variable gene in each cluster is shown.

The size of each AntiRef dataset, in number of sequences and as a fraction of the total input data, is shown in Fig. 1a. Notably, AntiRef100, which contains all unique sequences from the filtered OAS data, compressed the input dataset by over 25%. AntiRef90, which uses the least stringent clustering threshold, is 65% smaller than the input dataset. The compression of AntiRef90 is similar to that observed with UniRef50 (70%), indicating that the Ab-specific clustering thresholds used for AntiRef produce results proportionate to the general protein thresholds used by UniRef.

### 3.2 Distribution of cluster sizes

Cluster size frequencies in UniRef datasets follow a power law distribution, meaning the UniRef clustering approach effectively increases dataset diversity by collapsing highly similar sequences that are (in some cases, massively) over-represented. As a result, the general protein language model ESM-2 demonstrated marked improvement when trained with UniRef clusters rather than raw sequence data (Lin *et al.* 2022). Cluster size frequencies in each AntiRef clustering dataset showed a power law distribution (Fig. 1b), providing additional evidence that the benefits of using UniRef datasets in the general protein space can be replicated when using AntiRef datasets in Ab-specific contexts.

### 3.3 Genetic composition of AntiRef clusters

To determine the extent to which AntiRef clusters include different Variable (V) genes and/or V alleles, we sampled the 100 largest clusters from each AntiRef dataset and computed the fraction of each cluster encoding the single most common V allele in the cluster (Fig. 1c). As expected, the lower identity AntiRef datasets contain a more clusters for which alternative V genes are a substantial fraction of the total cluster. A similar experiment analyzing the complementarity determining region 3 (CDR3) length distributions for the same sample of clusters showed no variability in CDR3 length within any of the clusters.

## 4 Discussion and future work

Inspired by the usefulness of UniRef databases for various computational analyses of general proteins, AntiRef has been created to fill a similar role for Ab sequences. A series of AntiRef datasets have been generated using a nested clustering approach and Ab-specific identity thresholds. The uniqueness of antibodies within the general protein space necessitates the construction of Ab-specific resources like AntiRef. Proteins with high sequence similarity tend to have similar function, however, although similar Ab sequences are often functionally similar, a small number of differences in antigen recognition regions can result in highly divergent structural or functional properties. Additionally, the modular nature of Ab recombination means that tools and algorithms trained using general protein datasets may perform very well for the majority of the Ab protein encoded by these conserved, modular components but perform very poorly in the untemplated and often structurally labile antigen binding regions that determine Ab function.

By making available all code needed to completely reproduce AntiRef, we hope to encourage future work extending AntiRef beyond human antibodies to additional species and T cell receptors.

## Data Availability

All AntiRef datasets are available via Zenodo (Briney 2022) under the CC-BY 4.0 license, which matches the license under which OAS data are released. All code used to generate AntiRef is available via GitHub (github.com/briney/antiref) under the MIT license. We anticipate AntiRef updates will be released bi-annually, with the option for out-of-band updates when large or particularly interesting datasets become available. The AntiRef versioning scheme (current version: v2022.12.14) refers to the date on which sequences were retrieved from OAS.
